# Neurogenesis and Neuroinflammation in Dialogue: Mapping Gaps, Modulating Microglia, Rewiring Aging

**DOI:** 10.3390/cells15010078

**Published:** 2026-01-03

**Authors:** Masaru Tanaka

**Affiliations:** Danube Neuroscience Research Laboratory, HUN-REN-SZTE Neuroscience Research Group, Hungarian Research Network, University of Szeged, Tisza Lajos krt. 113, H-6725 Szeged, Hungary; tanaka.masaru.1@med.u-szeged.hu; Tel.: +36-62-342-847

**Keywords:** neurogenesis, neuroinflammation, aging brain, microglia, hippocampus, cognitive decline, Alzheimer disease, inflammasomes, epigenetics, translational research

## Abstract

**Highlights:**

**What are the main findings?**

- Five mechanistic gaps were defined that shape the neurogenesis–neuroinflammation dialogue in aging.
- Translational strategies such as imaging, immunomodulation, and glial reprogramming offer testable intervention pathways.

**What is the implication of the main finding?**

- Tuning immune and epigenetic environments may preserve or even restore neurogenic potential.
- An integrated roadmap links mechanistic precision to clinical innovation, aiming to delay cognitive decline.

**Abstract:**

**Background:** Aging brains are shaped by a persistent dialogue between declining neurogenesis and rising neuroinflammation. Neural stem cells progressively lose regenerative capacity, while microglia and astrocytes shift toward maladaptive states that erode synaptic plasticity and cognition. This convergence defines inflammaging, a slow yet relentless process that undermines resilience. However, the field remains hampered by critical gaps: incomplete mapping of microglial heterogeneity, poorly understood epigenetic scars from inflammasome signaling, lack of longitudinal data, unclear niche-specific immune mechanisms, and uncertain cross-species relevance. This review addresses these pressing barriers, aiming to transform fragmented insights into actionable strategies. **Summary:** I chart how neurogenesis and neuroinflammation operate in continuous dialogue, identify five major knowledge gaps, and evaluate strategies to reprogram this interaction. Approaches include longitudinal imaging, niche-focused immunomodulation, glial subtype reprogramming, brain-penetrant inflammasome inhibitors, and CRISPR-based epigenetic editing. Each strategy is mapped against translational potential, short-term feasibility, and long-term vision, with emphasis on how mechanistic precision can guide clinical innovation. **Conclusions:** Here I highlight that neurogenic potential is not entirely lost with age but may be preserved or restored by tuning immune and epigenetic environments. This review proposes a roadmap for reshaping the aging brain’s fate, offering mechanistically grounded strategies to delay cognitive decline. Beyond neurology, the work underscores a broader principle: by integrating cellular plasticity with immune modulation, science edges closer to re-engineering resilience across the lifespan.

## 1. Introduction

The brain remains a dynamic organ across the lifespan, continuously reshaped by the birth of new neurons in specialized niches such as the hippocampus [1,2]. Far from being a relic of development, adult neurogenesis enriches learning, memory, and emotional resilience, safeguarding adaptability in a changing environment [3,4]. Yet this plasticity is not inexhaustible. With aging, neurogenic output wanes, cognitive reserve diminishes, and vulnerability to neurodegeneration grows [5,6]. This tension between a system designed for renewal and its gradual attrition defines a central challenge for brain health, setting the stage for how neurogenesis and neuroinflammation intersect in the aging brain [7,8].

**Figure 1:**
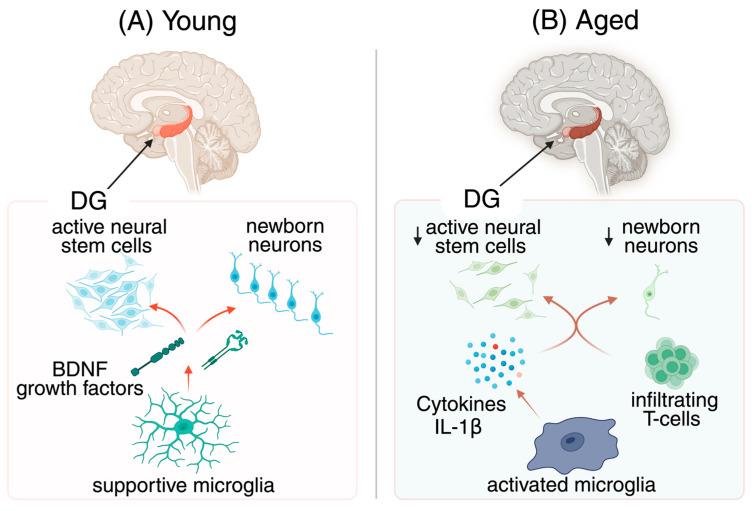
Neurogenesis–neuroinflammation dynamics across the lifespan. (**A**) Young: In the healthy young dentate gyrus (DG), abundant and actively cycling neural stem cells generate newborn neurons that successfully mature and integrate into local circuits. Supportive microglia maintain a trophic environment by releasing BDNF and other growth factors, which promote stem-cell activation and neuronal differentiation [26,27,28]. Arrows indicate pro-neurogenic signaling from microglia to stem cells and forward progression from stem cells to newborn neurons. (**B**) Aged: In the aged DG, stem-cell activation declines and fewer newborn neurons are produced. Microglia adopt reactive phenotypes in response to extrinsic cues (DAMPs, cytokines, peripheral immune signals, metabolic stress, BBB leakage) and intrinsic programs (epigenetic priming/innate immune memory, mitochondrial dysfunction, senescence-linked remodeling), and secrete pro-inflammatory cytokines such as IL-1β that suppress neurogenesis [12,21,24,29,30]. Arrows highlight the shift from supportive trophic signals to inflammatory cues and the resulting reduction in neuronal output. BDNF, brain-derived neurotrophic factor; DG, dentate gyrus; IL-1β, interleukin-1 beta. Created in Biorender. Tanaka, M. (2026) https://BioRender.com/pjz1qcu.

Aging is accompanied by a persistent, low-grade inflammatory state often termed inflammaging, a process distinct from acute infection yet equally influential in shaping brain health [9,10]. In this slow-burn process unfolding over years to decades, microglia gradually lose their homeostatic balance, adopting pro-inflammatory phenotypes that release cytokines and chemokines [11,12] (Figure 1). Astrocytes amplify this tone, shifting toward reactive states that erode trophic support and disrupt neuronal networks [13,14]. Vascular changes weaken the blood–brain barrier (BBB), while peripheral immune signals infiltrate and reinforce local inflammation [15,16]. Together, these subtle but enduring perturbations accumulate over decades, progressively altering cellular behavior and circuit resilience [17,18,19].

Across the aging hippocampus, two intertwined trajectories emerge: a steady decline in neurogenesis and a progressive rise in neuroinflammation [20,21] (Figure 1). Diminished neural stem cell activity and impaired maturation of adult-born neurons reduce pattern separation, flexibility in memory strategies, and mood regulation [22,23]. At the same time, microglia shift toward a proinflammatory state, releasing cytokines such as interleukin-1 beta (IL-1β) and TNF while offering reduced trophic support, thereby altering the niche [22,24]. Rather than separate phenomena, these arcs converge into a bidirectional dialogue in which inflammation curtails neurogenesis, and neurogenic failure amplifies vulnerability to inflammatory stressors [8,25].

Microglia operate as finely tuned gatekeepers of the neurogenic niche, shaping whether new neurons thrive or fail [26,31] (Figure 1). In youthful contexts, they clear apoptotic cells, sculpt synapses with precision, and secrete trophic factors such as brain-derived neurotrophic factor (BDNF) and IGF-1 that sustain progenitor proliferation and survival [26,32] (Figure 1A). Yet chronic inflammatory tone rewires their functions: cytokine release intensifies, complement-driven pruning accelerates, and phagocytic activity becomes biased toward eliminating viable cells [33,34]. This shift suppresses neurogenesis, disrupts circuit integration, and fosters vulnerability [31,35]. Crucially, youthful microglia are not merely less reactive; they are actively programmed toward pro-neurogenic states that are progressively lost with age [33,36].

Experimental models demonstrate that inflammatory insults sharply constrain adult hippocampal neurogenesis [37,38]. Acute lipopolysaccharide challenges, chronic peripheral inflammation, or autoimmune insults reduce progenitor proliferation, neuronal survival, and integration [39,40]. Conversely, targeted interventions ranging from pharmacological agents to trophic factors rescue aspects of neurogenesis by dampening microglial activation or restoring signaling cascades such as PI3K-Akt, ERK, or wingless-related integration site signaling pathway (Wnt)/β-catenin [41,42]. Yet evidence cautions against simple “anti-inflammation” strategies: both excessive suppression and prolonged activation can be deleterious [35,43]. Outcomes depend on timing, intensity, and niche context, underscoring the need for mechanistic precision in modulating pathways, microglial states, and local environments [8,44].

Despite mounting evidence, several gaps blunt causal inference and limit translation. Gap 1 is the scarcity of longitudinal, within-subject datasets that track how niche inflammation and neurogenesis co-evolve across aging, stress exposure, and recovery [45,46]. Gap 2 is that microglial and astrocytic heterogeneity is vast but still rarely mapped at neurogenic zones with sufficient spatial and temporal resolution [47,48]. Gap 3 is that inflammasome-linked innate immune memory may stabilize antineurogenic programs, but cell-type–specific persistence and reversibility remain unclear [49,50]. Gap 4 is that vascular, BBB, and peripheral immune contributions are often treated as background variables rather than measured drivers [51,52]. Gap 5 is that cross-species alignment remains underpowered, complicating inference about which mechanisms are shared versus model-bound [53,54].

This review sets out a playbook for rewiring the neuroimmune dialogue by linking mechanistic insight to translational strategy. I map unresolved gaps to actionable approaches: longitudinal neuroimmune imaging to capture temporal causality, niche-focused immunomodulation to tune local signals, and glial subtype reprogramming to restore supportive states or even generate new neurons. I highlight brain-penetrant nod-like receptor protein 3 (NLRP3) inhibitors and nucleic acid therapeutics as near-term strategies to break maladaptive IL-1β loops, while CRISPR-based epigenetic editing represents a longer-horizon tool to reset maladaptive chromatin programs. Together, these advances reframe therapeutic feasibility in aging.

Preserving a youthful neurogenic niche holds the promise of sustaining cognitive reserve, delaying neurodegeneration, and enhancing resilience across the lifespan [55]. Mechanistic advances reveal that both lifestyle factors and molecular interventions can counter inflammaging, rejuvenate progenitors, and restore plasticity [56]. The challenge is moving from associations to actionable strategies that align biology with translation [57]. This review charts that trajectory by first detailing the intertwined biology of neurogenesis and neuroinflammation, then interrogating five critical gaps that obscure causality, and finally evaluating emerging strategies with human applicability in view, guiding readers from concept to clinic-ready hypotheses.

Mechanistic advances reveal that both lifestyle factors and molecular interventions can counter inflammaging, rejuvenate progenitors, and restore plasticity [56]. The challenge is moving from association signals to testable mechanisms and actionable strategies that align biology with translation [57]. This review charts that trajectory by first detailing the intertwined biology of neurogenesis and neuroinflammation, then interrogating five critical gaps that obscure causality, and finally evaluating emerging strategies with human applicability in view, guiding readers from concept to clinic-ready hypotheses.

In this review, we use the term microglia primarily to denote yolk sac–derived parenchymal microglia residing within the brain parenchyma. However, several of the studies we discuss rely on broad myeloid markers such as Iba1 or CD11b, which do not fully discriminate parenchymal microglia from other tissue-resident brain macrophages including perivascular, meningeal, and choroid plexus macrophages or from infiltrating monocyte-derived macrophages. Where the original data do not clearly separate these populations, we therefore refer to microglia/macrophages and interpret the reported effects on neural stem and progenitor cells (NSPCs) as arising from mixed myeloid subsets rather than microglia alone. In contrast, when microglia are selectively targeted by genetic, pharmacological, or depletion approaches, we explicitly attribute the observed pro- or anti-neurogenic effects on NSPCs to parenchymal microglia. Causal inference should be confined to studies that employ microglia-selective perturbations; otherwise, the findings are best interpreted as reflecting niche-level inflammatory influences.

## 2. Neurogenesis and Neuroinflammation in the Aging Brain: An Overview

Adult mammalian brains retain a limited capacity for neurogenesis, confined mainly to the dentate gyrus (DG) of the hippocampus and the SVZ zone, where neural stem cells and NSPCs generate new neurons that integrate into existing circuits [58,59]. By contrast, in the neonatal brain NSPCs are distributed not only within these canonical neurogenic zones but also across additional regions such as the cerebral cortex, and these neonatal progenitors display molecular and functional traits that differ from adult NSPCs [60]. Similar to microglia, these NSPCs are not a uniform pool but encompass distinct subtypes with different degrees of quiescence, proliferative capacity, and lineage bias, particularly well characterized in the SVZ, where type B, C, and A cells have been delineated in both neonatal and adult brains [61,62]. This process diminishes with age, as stem cells adopt a primed, pro-inflammatory phenotype, releasing cytokines such as IL-1β and TNFα that impair progenitor proliferation and neuronal differentiation [63,64]. By contrast, anti-inflammatory and trophic factors like IL-4, IL-10, IGF-1, and BDNF promote neurogenesis [65,66]. The balance between these opposing signals shifts during “inflammaging,” when systemic immune mediators and infiltrating cells increasingly shape the neurogenic niche [63,67,68,69].

### 2.1. Adult Neurogenesis: Mechanisms and Age-Related Decline

Adult neurogenesis in the mammalian brain occurs primarily in two discrete regions, the subgranular zone of the hippocampus and the SVZ zone of the forebrain, where neural stem cells sustain lifelong plasticity by generating new neurons and glia [70,71]. Within these niches, stem cells undergo sequential steps of proliferation, lineage commitment, and differentiation into intermediate progenitors that ultimately mature into functional granule neurons or glial cells [72,73]. Newly generated neurons progress through migration, synaptic integration, and circuit incorporation, thereby reshaping hippocampal and olfactory networks while maintaining a dynamic balance between neuronal and glial lineages [74,75].

The regulation of adult neurogenesis reflects a delicate interplay between intrinsic genetic programs and extrinsic environmental cues [66,76]. Transcription factors and epigenetic mechanisms orchestrate lineage progression, guiding neural stem cells from quiescence toward neuronal or glial differentiation [58,77]. Simultaneously, the neurogenic niche provides trophic support, vascular inputs, glial signaling, and neuronal activity that sustain proliferation and integration [78,79]. Acting as a dynamic coordinator, the niche integrates these signals to preserve stem cell function and ensure a balanced neurogenesis output under physiological conditions [80,81].

Rodent studies consistently demonstrate that adult neurogenesis undergoes a steep decline with advancing age, marked by reduced progenitor proliferation and a shrinking contribution of new neurons to hippocampal circuits [82,83]. While neural stem cells persist, their output is curtailed by prolonged quiescence, asymmetric division, and intrinsic alterations such as diminished lamin B1 expression [84,85]. Equally decisive is the aging microenvironment: decreased trophic support, vascular dysfunction, and elevated TGF-β and inflammatory signaling constrain neurogenic potential [86,87]. These findings underscore that the decline is not due to progenitor loss but to niche deterioration, which restricts activation and differentiation despite preserved stem cell reservoirs [88,89,90].

Evidence from human postmortem and imaging studies indicates that hippocampal neurogenesis likely persists across adulthood, with several investigations detecting thousands of immature neurons in healthy individuals well into the eighth or even ninth decade [1,91]. Yet, other studies describe steep age-related reductions in proliferation and neurogenic markers, despite the continued presence of progenitor cells [22,92]. These conflicting findings are often attributed to methodological differences in tissue processing and marker detection [93,94]. The resulting uncertainty contrasts with rodent data, where neurogenic potential is retained but niche decline dominates, creating a translational dilemma that frames ongoing cross-species comparisons [92,95].

### 2.2. Neuroinflammation in Aging: Microglia and Beyond

The aging brain is characterized by a progressive remodeling of its immune landscape, where a state of low-grade but chronic neuroinflammation becomes a defining hallmark [96,97,98]. Central to this shift are microglia, the resident immune cells that gradually lose their homeostatic and reparative functions while adopting pro-inflammatory, neurotoxic phenotypes [11,12,99]. Hallmarks of aged microglia include altered transcriptomes, dystrophic morphology, impaired phagocytosis, and exaggerated cytokine release [11,100,101]. Yet microglia do not act alone; astrocytic immunosenescence and peripheral immune inputs further amplify inflammatory tone, contrasting sharply with the supportive environment of younger brains [102,103,104]. Astrocytes are not passive bystanders: with age they enter reactive programs (often described as A1/A2-like, but better viewed as a spectrum of reactive states) that reshape synaptic and stem-cell niches [13,105,106]. Reactive astrocytes release cytokines/chemokines and gliotransmitters (e.g., ATP, glutamate, D-serine) that can amplify inflammatory signaling and directly bias neural stem/progenitor cell quiescence, proliferation, and lineage decisions [26,107,108]. Through their endfeet at the blood–brain barrier, astrocytes act as gatekeepers of barrier permeability and immune-cell entry, while their metabolic coupling (lactate shuttling, lipid/cholesterol handling) can either buffer or fuel neuroinflammatory tone [107,108,109]. Microglia-to-astrocyte signals (e.g., IL-1–linked and complement-linked cues) promote astrocytic reactivity, whereas astrocyte-to-microglia signals can reciprocally tune microglial state, creating a local amplification loop [107,110,111]. In neurogenic regions, this astrocyte–microglia axis functions as a signal amplifier and gatekeeper that helps determine whether inflammatory episodes resolve with restored neurogenesis or consolidate into a chronically inhibitory niche [26,107,112].

Aged microglia are marked by dystrophic morphology, diminished phagocytic capacity, and transcriptional reprogramming that favors pro-inflammatory gene expression over reparative functions [11,113,114]. This deterioration is compounded by microglial priming, a process in which prior immune or metabolic challenges leave cells in a state of innate immune memory, heightening their responsiveness to subsequent insults [115,116,117]. Primed microglia release exaggerated amounts of cytokines such as IL-1β, IL-6, and TNFα, impairing synaptic plasticity and accelerating neurodegeneration [17,116,118]. Even when replaced experimentally, aged microglia retain their hyperreactivity due to niche-driven cues, underscoring how priming locks biases the aging brain into a maladaptive inflammatory state [114,119]. A key open question is timing: when do inflammatory and metabolic hits shift the niche from adaptive plasticity to persistent antineurogenic bias?

Beyond these structural and transcriptional shifts, chronic activation of inflammatory pathways—including NF-κB signaling and NLRP3 inflammasome activity—emerges as a central driver of aging-associated microglial dysfunction [120,121]. Their persistent activation sustains the release of IL-1β, IL-6, and TNFα, creating a hostile milieu that erodes neuronal survival and is linked to reduced adult neurogenesis in inflammatory and aging models [122,123,124]. These cytokines impair progenitor proliferation, bias glial differentiation, and disrupt synaptic plasticity, gradually shifting the neurogenic niche from supportive to inhibitory [8,125,126]. Importantly, mitochondrial dysfunction and oxidative stress further amplify NF-κB and NLRP3 activity, locking the system into a cycle of chronic inflammation that undermines regenerative capacity in the aging brain [124,127,128,129,130].

With advancing age, the blood–brain barrier becomes increasingly permeable, weakening its selective function and permitting infiltration of peripheral immune cells [16,131]. Among these, CD8^+^ T cells accumulate in neurogenic niches of aged mice and humans, where they release interferon-γ and other cytokines that suppress neural stem cell proliferation and neuronal differentiation [132,133]. Aged microglia further facilitate this process by secreting chemokines and remodeling the niche microenvironment, creating a feed-forward inflammatory loop [51,134]. In sharp contrast, the young brain maintains a largely anti-inflammatory, pro-neurogenic milieu, highlighting how immune remodeling with age tilts the balance away from regeneration toward chronic dysfunction [35,134,135].

### 2.3. Microglia–Neural Stem Cell Crosstalk

Microglia have emerged as central orchestrators of adult neurogenesis, engaging in a continuous dialogue with neural stem and progenitor cells that shapes every stage of the process [32,136,137]. Far from passive sentinels, they actively sculpt the neurogenic niche by phagocytosing apoptotic newborn cells, thereby maintaining homeostasis and determining which neurons survive to maturity [137,138]. Microglia also refine synaptic connections of adult-born neurons through selective pruning, ensuring proper integration into existing circuits [31,32,139]. Beyond these structural roles, their secretome exerts powerful influence, releasing context-dependent cues that either promote proliferation and differentiation or restrict neurogenesis, highlighting their dual capacity as nurturers or inhibitors [137,140].

In the young brain, microglia frequently adopt phenotypes that nurture rather than hinder neurogenesis [141,142,143]. By secreting trophic factors such as BDNF, IGF-1, and TGF-β, they stimulate neural stem cell proliferation, guide differentiation, and promote survival of newborn neurons [26,143,144]. Environmental enrichment and physical activity further enhance this supportive role, shifting microglia toward anti-inflammatory states that amplify plasticity and circuit integration [26,145,146]. M2-polarized microglia in particular foster neuronal differentiation and synaptic maturation, underscoring their capacity to translate systemic and local signals into pro-neurogenic outcomes [147,148,149]. This trophic partnership highlights microglia as crucial allies in sustaining hippocampal resilience early in life [26,27,142].

Aging and chronic stress profoundly disrupt microglia–neural stem cell interactions, and are associated with shifts toward pro-inflammatory, injury-responsive microglial programs that can become neurotoxicity-linked in specific contexts [29,115]. In this maladaptive phenotype, microglia secrete elevated levels of IL-1β, TNFα, and IL-6, which suppress NSC proliferation, reduce BDNF availability, and block the maturation of newborn neurons [12,30,99]. At the same time, microglia lose their phagocytic balance, leading to excessive or aberrant pruning that compromises neuronal survival and synaptic plasticity [11,99,150]. Impaired autophagy and metabolic dysfunction exacerbate these changes, and microglial priming—together with BBB alterations and peripheral immune inputs—can stabilize inflammatory niche states that progressively undermine regenerative potential [99,100,151].

Microglia–NSC crosstalk is mediated by finely tuned molecular pathways, with the C-X3-C motif chemokine ligand 1 (CX3CL1)–C-X3-C motif chemokine receptor 1 (CX3CR1) axis emerging as a central regulator of microglial activation, synaptic integration, and neurogenic support [28,152]. This signaling maintains microglial quiescence, limits cytokine release, and facilitates proper maturation of adult-born neurons, while its disruption impairs dendritic spine formation and neurogenesis [152,153,154]. Other modulators, including cytokines, chemokines, and extracellular vesicles (EVs), complement this dialogue by shaping microglial states and their influence on progenitors [155,156,157]. With aging, these pathways shift from protective to maladaptive, fostering chronic inflammation and reduced neurogenic output, thereby highlighting their therapeutic relevance (Table 1), which cites the primary evidence base underlying each pathway [28,158].

## 3. Critical Gaps in Current Knowledge

Building on the five gaps introduced above, the following sections align mechanistic uncertainties with practical experimental and translational strategies (Table 2).

### 3.1. Gap 1—Region-Specific Microglial Diversity in Aging

**Figure 2:**
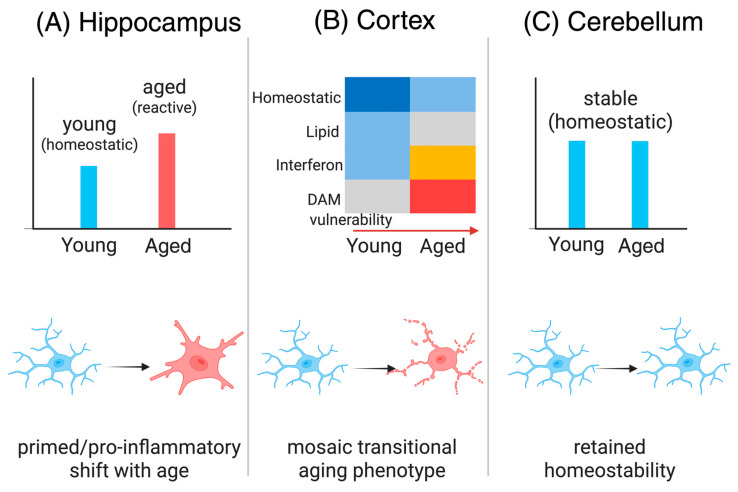
Region-specific microglial diversity and aging. (**A**) Hippocampus: Hippocampal microglia shift from a predominantly homeostatic profile in young animals to a primed, pro-inflammatory state with aging. This is illustrated by increased reactive microglial signatures and elevated inflammatory markers, reflecting the heightened vulnerability of the neurogenic dentate gyrus (DG). The upward arrow indicates an age-related rise in priming and inflammatory activation [24,29,116]. (**B**) Cortex: Cortical microglia exhibit a mosaic, transitional aging phenotype. The heatmap summarizes relative transcriptional activities—homeostatic, lipid-associated, interferon-responsive, and DAM (disease-associated microglia) vulnerability—across young and aged states. Color gradients and the horizontal age-arrow illustrate how these signatures shift heterogeneously with age, highlighting multiple partially overlapping microglial subtypes rather than a uniform transition [47,165]. Dark blue—high homeostatic activity; Light blue—moderate homeostatic activity; Grey—lipid-associated activity; Yellow—interferon-responsive activity; Red—high DAM-vulnerability activity (**C**) Cerebellum: Cerebellar microglia largely preserve a stable, homeostatic phenotype across aging. Bar plots and conceptual illustrations show minimal change in activation markers, and microglia remain predominantly ramified with limited shift toward inflammatory states [166,167]. Overall interpretation: Microglial aging is regionally diverse. Hippocampal microglia tend toward primed, pro-inflammatory profiles; cortical microglia adopt mixed and transitional states; and cerebellar microglia maintain homeostability. This heterogeneity reveals that aging does not uniformly reprogram microglia across the brain and underscores the need for spatially tailored therapeutic strategies targeting region-specific vulnerabilities. DAM, disease-associated microglia. Created in Biorender. Tanaka, M. (2026) https://BioRender.com/vu0jdxr.

Microglia are increasingly recognized as a heterogeneous population whose identities vary across brain regions rather than fitting a uniform template [47]. Transcriptomic and single-cell profiling studies reveal distinct gene expression and morphological features in microglia from the cortex, hippocampus, cerebellum, and other regions, with some subsets tuned toward surveillance and others toward immune activation [164]. These region- and cell-type relationships are summarized in Figure 2. Aging accentuates these differences, reshaping transcriptional signatures in a region-dependent manner and amplifying selective vulnerabilities [47]. This diversity represents a critical yet underexplored determinant of brain aging and resilience [97]. These region- and cell-type relationships are summarized in Figure 2.

Aging imprints distinct signatures on microglia across brain regions, revealing striking contrasts in phenotype and function [47]. In the hippocampus, transcriptomic analyses show upregulation of adhesion and motility genes, aligning with greater sensitivity to inflammatory and metabolic stress, while cerebellar microglia appear comparatively stable [47] (Figure 2A). Experimental challenges further underscore this diversity: TNFα or systemic LPS elicit robust and prolonged activation in hippocampal microglia, yet only muted responses in other regions [168]. These findings highlight that microglial aging is not uniform [47]. What remains unresolved is how such region-specific shifts shape neuronal survival, plasticity, and ultimately cognitive aging [97].

**Figure 3:**
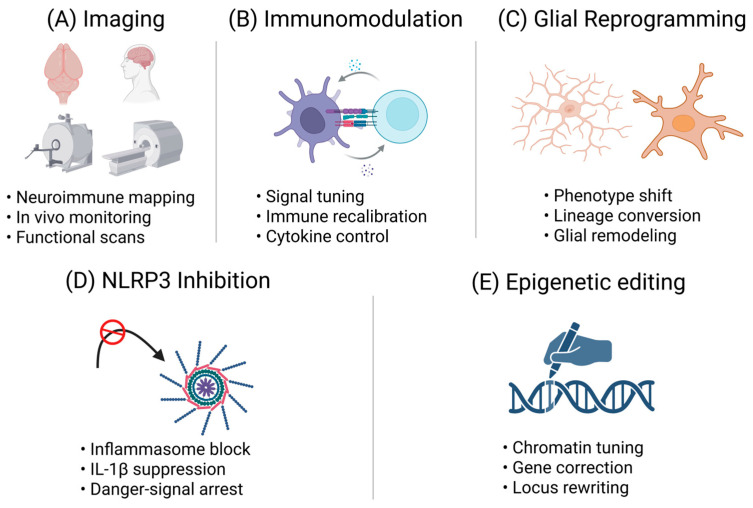
Neuroimmune interventions—from mechanism to therapeutic direction. (**A**) Imaging: Depicts neuroimmune mapping tools used for diagnostic and analytical purposes. The bullets refer to: Neuroimmune mapping—visualization of microglial and inflammatory states; In vivo monitoring—longitudinal tracking of neurogenic and neuroimmune changes; Functional scans—PET/MRI measures linking immune activity to neurogenic output. These modalities assess, rather than modify, biological pathways [216,217,218]. (**B**) Immunomodulation: Illustrates strategies that recalibrate maladaptive immune activity. The bullets indicate: Signal tuning—adjusting microglial or cytokine signaling thresholds; Immune recalibration—restoring balanced immune tone; Cytokine control—targeted modulation of IL-1β, TNF, IL-6, or related mediators [24,29]. (**C**) Glial Reprogramming: Represents approaches that shift glial cell identity or function. Bullets correspond to: Phenotype shift—moving microglia or astrocytes into supportive states; Lineage conversion—direct astrocyte-to-neuron or glia-to-neuron reprogramming; Glial remodeling—restructuring glial networks to enhance neurogenesis [192,219,220]. (**D**) NLRP3 Inhibition: Highlights suppression of inflammasome-mediated inflammation. Bullets denote: Inflammasome block—direct inhibition of NLRP3 assembly/activation; IL-1β suppression—reduction in downstream pro-inflammatory cytokine release; Danger-signal arrest—prevention of upstream triggers driving chronic activation [221,222,223,224,225,226]. (**E**) Epigenetic Editing Shows locus-specific chromatin tuning tools. The bullets signify: Chromatin tuning—modifying histone or DNA accessibility at inflammatory loci; Gene correction—targeted suppression or activation of disease-relevant genes; Locus rewriting—durable transcriptional reprogramming without DNA cleavage [227,228,229,230,231,232]. This figure provides a modular overview of five neuroimmune intervention strategies spanning monitoring, immune recalibration, glial engineering, inflammasome blockade, and epigenomic control. Together, they outline mechanistic entry points for preserving or restoring adult neurogenesis. Imaging modalities function strictly as diagnostic and analytical tools rather than interventions, supporting evaluation of therapeutic effects on neurogenic and inflammatory dynamics. IL-1β, interleukin-1 beta; NLPR3, NLR family pyrin domain containing 3. Created in BioRender. Tanaka, M. (2026) https://BioRender.com/073ljub.

Neurogenic regions such as the DG and SVZ zone rely on close interactions between neural stem cells and local microglia, yet whether these microglia display unique aging trajectories remains an unresolved question [21] (Figure 3C). Evidence suggests that niche-resident microglia have specialized roles, from supporting neuroblast migration to modulating survival signals [169]. With age, however, these populations undergo positional remodeling and progressive activation that may create antineurogenic environments [134]. What is missing is systematic mapping of their transcriptional and functional states across the lifespan [21]. Without such resolution, it is difficult to disentangle whether neurogenesis declines mainly from local niche deterioration or reflects broader systemic shifts in microglial aging [170].

Resolving how microglial diversity shapes neurogenic decline carries profound implications for therapy [171]. If hippocampal microglia are particularly prone to adopting pro-inflammatory, anti-neurogenic profiles with aging, while SVZ microglia preserve more supportive functions, this could help explain selective vulnerabilities in cognition and neurodegeneration [172]. Such distinctions suggest that interventions need not silence microglia globally but instead target maladaptive phenotypes in specific regions [48]. High-resolution profiling of microglial states in neurogenic versus non-neurogenic regions will therefore be critical [173]. These insights could enable tailored strategies that restore hippocampal neurogenesis locally while preserving beneficial immune surveillance elsewhere [174].

### 3.2. Gap 2—Inflammasome-Driven Epigenetic Alterations

Persistent activation of the NLRP3 inflammasome has emerged as a hallmark of brain aging, shaping a chronic inflammatory environment that disrupts neuronal and stem cell homeostasis [175]. Unlike acute responses, which are transient and protective, aged microglia remain locked in an overactivated state, driving continual secretion of IL-1β and interleukin-18 (IL-18) [176]. This sustained output fuels neuroinflammation, amplifies synaptic dysfunction, and accelerates neuronal loss [175]. Evidence from Alzheimer’s disease (AD) and other age-related contexts shows that NLRP3 activation is maintained by metabolic stressors and amyloid accumulation, marking it as a central instigator of the pro-inflammatory niche characteristic of the aged brain [177].

A growing body of evidence shows that aged microglia carry an epigenetic memory of past inflammatory encounters, leaving behind enduring “scars” that sustain maladaptive activity [178]. Hypomethylation of the IL-1β promoter, for instance, maintains excessive cytokine release in aging brains and drives persistent neuroinflammation [178]. Such chromatin-based reprogramming distinguishes transient immune responses from long-lasting dysfunction [178]. Neural stem cells in inflamed niches may undergo similar repressive modifications at pro-neurogenic loci, reducing their regenerative potential [179]. Parallels with hematopoietic stem cells, where chronic inflammasome signaling reshapes enhancer accessibility, underscore how inflammation imprints itself epigenetically to constrain stem cell function across tissues [180].

Whether inflammasome signaling directly reshapes the epigenome of neural stem cells in the DG or SVZ zone remains an unresolved question [181]. NSCs in these regions may acquire repressive chromatin marks that blunt their regenerative responses long after inflammatory cues dissipate, yet systematic evidence is lacking [181]. Do aged NSCs inherit such ‘epigenetic scars,’ stabilizing diminished neurogenic potential? If so, these marks may lower the ceiling of repair even after inflammatory cues subside, making full rejuvenation harder without targeted resetting [181]. Current studies describe epigenetic regulation in adult NSCs and chromatin remodeling during aging, but they rarely examine inflammasome-driven mechanisms [77]. Without detailed chromatin and transcriptomic maps of inflamed niches, the link between persistent inflammation and neurogenic failure remains speculative [181].

If inflammasome activity imprints lasting epigenetic scars on neural stem cells, these changes could persistently dampen neurogenesis and lower the ceiling of regenerative responses, even after inflammatory cues subside [182,183]. However, studies of ischemia and stroke in rodents and humans demonstrate that locally activated, injury-responsive stem cells with reprogrammed phenotypes can still be recruited within damaged areas and contribute to neurogenesis [184,185]. Thus, rather than an absolutely “locked” state, inflammasome-driven epigenetic memory may shift neural stem cells along a continuum of reduced but still reactivatable potential, with important implications for how we design interventions to reset the niche.

### 3.3. Gap 3—Longitudinal Dynamics of Neuroimmune Interactions

Most studies examining the interplay between neuroinflammation and neurogenesis rely on static snapshots, typically contrasting young and old animals or measuring endpoints after an inflammatory insult [186]. While such designs capture broad differences, they cannot reconstruct dynamic trajectories or reveal causal order [187]. It remains unclear whether inflammatory changes precede neurogenic decline, arise in parallel, or follow as a secondary consequence [186]. Cross-sectional single-cell and epigenomic studies have enriched our understanding of cell states, yet they provide only frozen moments in time, leaving the temporal choreography of neuroimmune aging unresolved [188].

As outlined in Section 2.2, a central unresolved issue is timing—when inflammatory and metabolic perturbations transition from adaptive responses to persistent antineurogenic bias. Addressing this requires within-subject, longitudinal approaches that can resolve sequence and causality rather than relying on cross-sectional snapshots.

Closing the temporal gap will require methodological advances that move beyond static measures [189]. Longitudinal in vivo imaging of both neurogenesis and neuroinflammation, coupled with emerging PET tracers, offers one promising path [190]. Parallel development of peripheral and central biomarkers, alongside chronic experimental paradigms rather than acute LPS challenges, is equally critical [191]. Ultimately, integrated strategies that link molecular, cellular, and systems-level dynamics are needed to capture how neuroimmune interactions unfold across the lifespan [192].

Clarifying the temporal sequence between inflammation and neurogenesis is pivotal for understanding brain aging [21]. If chronic inflammation proves to be a driver, consequence, or both in neurogenic decline, this will fundamentally reshape strategies for preserving neural plasticity [193]. Untangling this interplay is therefore essential for precision approaches that safeguard neurogenic capacity across the lifespan and ultimately inform how we design therapies to maintain cognition and resilience in aging [194].

### 3.4. Gap 4—Niche-Specific Immune Mechanisms

The subgranular zone (SGZ) of the hippocampus and the SVZ of the lateral ventricles form highly specialized neurogenic niches, distinct from the broader brain parenchyma [79]. These microenvironments bring together neural stem cells, progenitors, astrocytes, microglia, endothelial cells, and, in aging, even infiltrating immune cells [79]. Yet, despite advances in transcriptomic and proteomic profiling, we still lack a clear map of which immune and inflammatory signals within these niches directly regulate stem cell activity [195]. Equally unresolved is whether resident or infiltrating immune cells dominate in suppressing neurogenesis during aging [195].

Growing evidence implicates inflammatory cues as major inhibitors of neurogenesis within the SVZ and subgranular zones [195]. Microglial-derived cytokines such as IL-1β, TNFα, and IL-6 consistently emerge as candidates, while monocyte infiltration and CD8+ T cell activity in the SVZ have also been linked to reduced neurogenic potential [132]. Yet causality and relative contributions remain unresolved [196]. Systemic inflammation further complicates matters by altering blood–brain barrier integrity and selectively reshaping niche immune composition, but the permeability and vulnerability of these sites are still poorly defined [197].

Part of this uncertainty reflects limitations of the current in vivo toolkit for dissecting NSPC–microglia/macrophage interactions. There are still no widely adopted approaches that suppress either NSPCs or microglia/macrophages in a fully selective and temporally precise manner in the intact brain. Pharmacological and toxic paradigms such as CSF1R inhibitors or liposomal clodronate can deplete microglia/macrophages, but they often produce incomplete or transient ablation, affect perivascular and meningeal brain macrophages as well as peripheral myeloid cells, and may indirectly influence NSPC proliferation and survival [198]. Similarly, strategies used to inhibit NSPCs in vivo typically impact other dividing glial and progenitor populations. As a result, changes in adult neurogenesis observed in these models cannot be attributed unequivocally to microglia versus other macrophage populations or to NSPCs themselves, underscoring the need for refined, cell-type–specific genetic and chemogenetic approaches to define causal relationships.

Not all immune influences within the neurogenic niche are detrimental [136]. Signals such as TGF-β, IL-10, IGF-1, and CX3CL1 are increasingly recognized as protective factors that can sustain or even restore neurogenesis [199]. Yet whether these mediators act in a niche-specific manner and how their decline contributes to age-related collapse of neurogenic capacity remain unanswered questions [200]. It is also unclear whether immune checkpoints or anti-inflammatory feedback loops normally shield stem cells from inflammatory stress but fail with aging, leaving the niche vulnerable to irreversible dysfunction [200].

The idea of a “niche immunome” has emerged as a powerful framework to decode the immune and inflammatory signals that shape neurogenic niches [201]. Single-cell and spatial transcriptomic approaches now allow systematic profiling of the SGZ and SVZ across age, revealing immune pathways that bulk parenchymal studies cannot resolve [132]. Such resolution is crucial for distinguishing local immune regulation of neural stem cells from generalized brain inflammation, and for uncovering niche-specific vulnerabilities that may define regenerative potential in aging [202].

Bridging neuroimmunology with regenerative neuroscience requires moving beyond broad immunosuppression toward niche-specific interventions [203]. Without precise insight into the immune circuits of the SGZ and SVZ, therapies risk silencing protective signals while failing to restore neurogenesis [203]. Evidence from aging models shows that targeted modulation of microglia, T cells, or cytokine pathways can rejuvenate neurogenic capacity, underscoring the therapeutic promise of restoring local immune balance [114]. Defining these mechanisms positions the niche immunome as a critical frontier for precision interventions in brain aging [203].

### 3.5. Gap 5—Translational and Cross-Species Disconnects

Most of what we know about the interplay between neuroinflammation and neurogenesis comes from rodent studies, yet rodents differ profoundly from humans in biology, lifespan, and environment [204]. Mechanisms that restore neurogenesis or cognition in mice often fail in clinical settings because molecular programs, immune responses, and even circadian rhythms diverge across species [101]. Human microglia show distinct transcriptional heterogeneity, and adult neurogenesis itself is limited and debated in humans compared with rodents [205]. This translational disconnect remains a central barrier, slowing progress from mechanistic insight to therapies that could counteract age-related cognitive decline [204].

Rodents display strikingly robust adult neurogenesis, both in the hippocampal DG and in the SVZ–olfactory bulb pathway, where thousands of new neurons are continually produced and integrated [206]. In contrast, humans lack meaningful SVZ-driven olfactory bulb neurogenesis, and hippocampal neurogenesis, though reported, is modest, controversial, and appears to decline with age [204]. Some studies suggest persistence across the lifespan, while others argue it is virtually absent in adulthood [95]. This lack of consensus complicates translational efforts, as strategies that reliably boost rodent neurogenesis—such as environmental enrichment, exercise, or pharmacological interventions—may have little impact in humans [207]. Without resolving this debate, applying pro-neurogenic therapies clinically remains fraught with uncertainty [208].

Human neuroinflammation diverges markedly from rodent models, complicating translational efforts [209]. Single-cell studies reveal that while core microglial programs are conserved, human microglia exhibit greater transcriptional heterogeneity, unique complement and phagocytic modules, and a baseline preactivated state not mirrored in rodents [205]. Moreover, human immune aging is shaped by lifelong infections, systemic comorbidities, and lifestyle exposures absent in laboratory animals, producing compounded inflammatory stress [100]. These differences raise concerns that interventions restoring neurogenesis in mice, such as exercise, cytokine modulation, or small molecules, may not yield comparable benefits in the aged human brain, underscoring the need for human-specific models and biomarkers [210].

Closing the translational gap requires models that capture the complexity of the human neuroimmune environment more faithfully than rodents [211]. Non-human primates offer closer physiology, yet complementary systems such as human brain organoids and induced pluripotent stem cell (iPSC)-derived microglia–NSC co-cultures now provide scalable and mechanistically precise tools [212]. These platforms permit interrogation of key pathways like NLRP3 or CX3CR1 in human-relevant contexts, while enabling controlled testing of immunomodulatory and pro-neurogenic therapies [211]. By combining primate studies with organoid and chip-based systems, researchers can generate clinically predictive insights that accelerate the translation of neuroimmune discoveries into interventions for aging-related decline [210].

Without human-specific insight, immunomodulatory therapies risk being blunt instruments, either failing to restore neurogenesis or disrupting essential immune functions [124]. Bridging species differences is therefore indispensable. By refining targets within human-relevant systems, interventions can be designed to preserve or even rejuvenate neurogenic capacity in aging, offering a path to meaningfully rewire the brain’s fate in clinical reality [213,214,215].

## 4. Strategies and Emerging Approaches to Bridge the Gaps

Having outlined the critical knowledge gaps, this section discusses five key strategies to address these gaps and modulate the neuroimmune dialogue for therapeutic benefit. Each subsection corresponds to a specific strategy highlighted in the abstract (longitudinal neuroimmune imaging, niche-focused immunomodulation, glial subtype reprogramming, brain-penetrant NLRP3 inhibition, and CRISPR-based epigenetic editing). For each approach, I will describe the concept, provide examples of current research or tools, and discuss how it can help fill one or more of the gaps identified in Section 3. I will also comment on the feasibility and timeline: which strategies are nearer-term vs. longer-term, and how they could be implemented in animal models or clinically (Figure 3).

### 4.1. Longitudinal Neuroimmune Imaging

While imaging modalities such as [^18^F]FLT-PET and TSPO-PET do not act as therapeutic interventions, they provide crucial diagnostic and monitoring capabilities (Figure 3A). These tools track neurogenesis, neuroinflammation, and treatment response in vivo, thereby supporting and enhancing the evaluation, optimization, and stratification of true interventional strategies. Longitudinal neuroimmune imaging is emerging as a transformative approach to address critical gaps in our understanding of brain aging [233]. It directly tackles Gap 3 by moving beyond cross-sectional “snapshot” methods, enabling dynamic monitoring of neurogenesis and neuroinflammation over time within the same subject [234]. Such temporal resolution can reveal whether surges in microglial activation precede, coincide with, or follow changes in neural stem cell activity [233,235]. Equally important, this strategy advances Gap 5 by providing non-invasive, cross-species applications through PET, magnetic resonance imaging (MRI), and two-photon microscopy [236]. By linking mechanistic insights from animal models to clinically relevant biomarkers in humans, it strengthens translational bridges [236,237].

Two-photon microscopy has revolutionized animal neuroimaging by enabling real-time, longitudinal observation of microglia–neuron interactions in the hippocampal neurogenic niche [238]. Using chronic cranial windows, researchers can track the same cells over weeks, revealing processes such as synaptic pruning, phagocytosis, and modulation of neural stem cell activity [238,239]. Fluorescent reporters and genetic labeling strategies further enhance cellular specificity, allowing precise mapping of immune–neural interactions [240]. These methods provide unmatched mechanistic insight into how inflammation and neurogenesis co-evolve, yet they remain invasive, limited to small animals, and restricted in imaging depth [241]. Despite these constraints, animal imaging offers critical proof-of-concept data that inspire translational strategies in humans [241,242].

Positron emission tomography and magnetic resonance imaging have become indispensable for longitudinal neuroimmune imaging, spanning both preclinical and clinical domains [243]. Translocator protein 18 kDa (TSPO)-PET is the most established approach for visualizing activated microglia, yet interpretation is hampered by low specificity, multicellular expression, and genetic polymorphisms that affect ligand binding [244]. To overcome these limitations, experimental PET tracers such as [^18F]FLT have been explored for labeling proliferating cells, with proof-of-concept studies showing that inhibiting tracer efflux enables detection of neurogenesis in vivo [216,243]. MRI provides a crucial complement, offering high-resolution structural measures such as hippocampal atrophy and functional connectivity readouts [245]. Together, PET and MRI form a translational bridge, linking cellular-level processes to human biomarkers and paving the way for therapeutic monitoring [246,247,248,249].

Next-generation imaging strategies are reshaping how we study neuroimmune dynamics. New PET tracers are being designed to distinguish between pro-inflammatory and anti-inflammatory microglial states, with promising targets such as P2 × 7R and P2Y12R offering phenotype-specific resolution [217,250]. Other tracers aim to directly label neurogenic processes or capture early astrocytic responses [217,251,252]. Hybrid modalities like PET/MR and dual-modal probes enhance spatial and molecular precision, while computational approaches including radiomics and machine learning refine signal interpretation and predict outcomes [253]. Integrating imaging readouts with peripheral biomarkers from blood or cerebrospinal fluid (CSF) promises a multimodal framework that could accelerate translation toward clinically actionable neuroimmune biomarkers [254].

The future of longitudinal neuroimmune imaging hinges on overcoming key technical and conceptual barriers [190]. New PET tracers are being developed to distinguish pro- and anti-inflammatory microglial phenotypes or to directly visualize neurogenic processes, promising greater specificity than TSPO-based tools [218]. Hybrid modalities such as PET/MRI enable integration of molecular and structural data, while computational approaches including radiomics and machine learning refine interpretation and enhance predictive power [255,256]. Pairing imaging with peripheral biomarkers from blood or CSF offers a multimodal strategy that could transform neuroimmune profiling, but achieving reliable, clinically translatable applications will require coordinated innovation and rigorous cross-species validation [190,257].

### 4.2. Niche-Focused Immunomodulation

Niche-focused immunomodulation refers to strategies that directly target the immune microenvironment of neurogenic regions such as the hippocampal DG and the SVZ zone, offering a sharp contrast to broad systemic immunosuppression [258] (Figure 3B). These niches are not only central to sustaining neurogenesis but also uniquely accessible for precision therapies [258]. Localized approaches include intranasal delivery of cytokines that preferentially concentrate in ventricular areas, biomaterials or hydrogels engineered for sustained release of modulators, and blood–brain barrier-permeable compounds that accumulate within neurogenic zones [259]. Such strategies have shown that tailoring immune signals at the site of neural stem cell activity can stimulate neurogenesis while minimizing systemic risks [258]. By focusing interventions where they are most needed, niche-targeted approaches provide a rational and clinically appealing pathway to restore or preserve brain plasticity in aging and disease [258,260].

Aged and inflamed neurogenic niches often recruit CD8+ T cells and monocytes that secrete interferon-γ and other inhibitory factors, directly suppressing stem cell proliferation and neurogenesis [132]. Neutralizing these detrimental influences has emerged as a promising strategy to protect niche integrity [261]. Approaches include preventing immune cell entry with antibodies targeting adhesion molecules like CD44, or blunting their effects through cytokine neutralizers such as IL-8 blockade. Experimental work shows that anti-inflammatory agents, including indomethacin and minocycline, can preserve hippocampal neurogenesis during inflammatory insults, underscoring the therapeutic potential of immune blockade [262]. While systemic immunosuppression risks broad deficits, restricting these strategies to the niche could selectively alleviate inhibitory signaling without impairing host defenses [132]. Such focused modulation offers a rational pathway to counter age- and disease-associated immune pressures while preserving the regenerative capacity of the brain [132].

Enhancing pro-neurogenic immune signals represents a complementary strategy to blocking detrimental drivers, aiming instead to amplify reparative pathways within neurogenic niches [136]. Skewing microglia toward an “M2-like” phenotype through IL-4, IL-13, TGF-β mimetics, or nanomaterial-based modulators has shown proof-of-concept benefits, improving neurogenesis in models of aging, injury, and neurodegeneration [263]. Beyond immune skewing, engineered astrocytes or transplanted neural stem cells can be programmed to secrete protective cytokines and trophic factors, creating self-sustaining pro-regenerative feedback loops [264]. Biomaterials and hydrogels further extend these approaches by providing sustained, localized release of immune modulators within the hippocampus or SVZ [265]. Such strategies capitalize on the unique accessibility of neurogenic zones to reprogram niche immunity from within [136]. By strengthening protective cues locally, rather than relying on systemic administration, therapies may more effectively counter the age-related decline of neurogenesis while minimizing adverse immune suppression [136].

Implementing niche-focused immunomodulation requires overcoming significant technical hurdles but also opens remarkable translational opportunities [88,266]. Precision delivery systems such as focused ultrasound can transiently open the blood–brain barrier in hippocampal or SVZ regions, enabling local administration of gene vectors, cytokines, or antibodies with reduced systemic spillover [267]. Engineered stem cell grafts or EVs offer additional routes to sustain protective immunomodulation directly within the niche, while gene therapy vectors can be tailored for long-term expression of pro-neurogenic signals [268]. Challenges remain, including achieving delivery accuracy, sustaining therapeutic effects, and accounting for heterogeneity across neurogenic zones [203]. These considerations tie closely to Gap 1, highlighting regional microglial diversity, and Gap 5, emphasizing cross-species disconnects that complicate translation [203]. By integrating innovative tools with human-relevant models, niche-focused immunomodulation emerges as a therapeutic bridge, transforming mechanistic insights into precision strategies to rejuvenate neurogenesis in aging brains [203].

### 4.3. Glial Subtype Reprogramming

Glial subtype reprogramming represents a bold therapeutic paradigm in which resident glia are reshaped to foster neural repair and adult neurogenesis [269] (Figure 3C). Two main strategies define this field. Phenotypic reprogramming focuses on restoring dysfunctional microglia or astrocytes to neuroprotective states, for example by inhibiting inflammatory cascades or promoting cross-talk that favors protective cytokine release [270]. Lineage reprogramming goes further, converting astrocytes into neurons or neural progenitors through transcription factors such as NeuroD1, DLX2, or Neurog2, or with small-molecule cocktails capable of inducing neuronal fates in vivo [219]. Together, these approaches seek to remodel the neurogenic niche, counteract age-related decline, and generate new avenues for brain rejuvenation rooted in cellular plasticity [266].

Phenotypic reprogramming of microglia has emerged as a compelling strategy to restore neurogenic potential within the hippocampal niche [271]. Central to this approach is the inhibition of NF-κB and related inflammatory cascades, which drive the release of cytokines that suppress neural stem cell proliferation [272]. Pharmacological interventions such as indole derivatives, natural compounds like mangiferin or costunolide, and small molecules targeting PI3K–Akt or Nrf2 signaling have successfully reduced pro-inflammatory activity while promoting anti-inflammatory/trophic microglial programs (often operationalized by IL-4–linked signatures) [122]. Similarly, exosome-based delivery of miR-124 or growth factors such as FGF1 rejuvenated microglial transcriptomes and enhanced neurogenesis [162]. Experimental evidence demonstrates that these interventions not only dampen pathological inflammation but also improve hippocampal neurogenic output, leading to cognitive and behavioral recovery in stress, injury, and neurodegenerative models [271].

Lineage reprogramming of astrocytes has revealed an extraordinary potential to regenerate neurons within damaged or aged brains [273]. Breakthrough studies demonstrate that transcription factors such as NeuroD1, DLX2, or Neurogenin-2 can directly convert reactive astrocytes into functional neurons in vivo, with newly generated cells integrating into local circuits and restoring behavioral function after injury or stroke [219]. Complementary approaches employ cocktails of small molecules to reprogram astrocytes into neurons or progenitor-like cells without viral vectors, offering a more clinically attractive route [274]. Both genetic and chemical strategies have produced proof-of-concept evidence that even reactive astrocytes in diseased or inflamed contexts can be redirected toward a neuronal fate [275]. These advances suggest that lineage reprogramming may one day augment or replace lost neurogenesis, transforming astrocytes into reservoirs of neuronal replacement [276].

Glial subtype reprogramming provides a bold strategy to tackle Gap 1, the challenge of regional microglial diversity, and Gap 2, the influence of inflammasome-driven epigenetic alterations [34]. By restoring homeostatic or neuroprotective microglial states, interventions counteract inhibitory cytokine cascades that suppress neural stem cells [277]. At the same time, lineage reprogramming of astrocytes into neurons or progenitors enlarges the neurogenic reservoir, directly compensating for age-related decline [266]. This dual action both mitigates maladaptive immune signaling and boosts neuronal output, reframing glia not as barriers but as therapeutic substrates for rejuvenating neurogenic niches [278].

Glial subtype reprogramming faces formidable but surmountable translational challenges [278]. Precision of delivery remains paramount, as viral vectors and gene editing tools pose risks of off-target effects, immune responses, and uncontrolled proliferation [279]. The heterogeneity of niches adds further complexity, demanding context-sensitive strategies rather than blanket interventions [280]. Innovative technologies such as CRISPR-based regulation, hydrogel-rationed delivery systems, and inducible gene circuits offer avenues for safer, more controlled reprogramming [281]. Ultimately, this approach represents a bold therapeutic frontier: the potential to “rewrite” the aging brain’s fate by generating neurons from glia and restoring supportive immune states within neurogenic niches [269].

### 4.4. Brain-Penetrant NLRP3 Inflammasome Inhibitors

The NLRP3 inflammasome has emerged as a central orchestrator of chronic neuroinflammation, with microglial activation driving sustained release of IL-1β that disrupts neural stem cell function and impairs neurogenesis [282] (Figure 3D). While systemic inhibition of this pathway shows anti-inflammatory promise, the distinct challenge in brain disorders lies in achieving effective suppression within the central nervous system (CNS) [221]. Small-molecule inhibitors capable of crossing the blood–brain barrier, such as nlrp3 inflammasome inhibitor mcc950 (MCC950) and newer candidates like NT-0796 or ASP0965, represent a breakthrough class [221]. By directly targeting microglial inflammasome activity in situ, these compounds address age- and disease-related priming of neuroinflammation that perpetuates cognitive decline and accelerates neurogenic failure [222].

MCC950 has served as the prototypical NLRP3 inhibitor, demonstrating consistent ability to cross the blood–brain barrier and attenuate microglial activation across diverse models of stress, injury, and neurodegeneration [223]. By suppressing caspase-1 activation and IL-1β release, it preserves neural progenitor proliferation and mitigates cognitive decline in contexts such as AD, stroke, traumatic brain injury (TBI), and depression-like states [224]. Building on this foundation, newer derivatives such as NP3-253 and novel bicyclic scaffolds have been designed for improved CNS penetration, stability, and potency [225]. Preclinical studies with these compounds confirm that inflammasome inhibition protects hippocampal neurogenesis, underscoring the therapeutic promise of this mechanistically targeted approach [283].

New brain-penetrant NLRP3 inhibitors such as NT-0796 and BGE-102 are advancing into early clinical trials, marking a pivotal step in translating inflammasome biology into therapy [221]. These compounds demonstrate robust CNS exposure and have been shown to lower neuroinflammatory biomarkers in humans, offering a promising route to intervene in age-related cognitive decline and mild cognitive impairment [221,284]. By disrupting IL-1β–driven feedback loops, they directly address Gap 2, mitigating inflammasome-driven epigenetic alterations that lock microglia into pro-inflammatory states [285]. At the same time, their clinical development speaks to Gap 5, bridging preclinical insights with druggable, human-relevant strategies aimed at rejuvenating neurogenic niches [286].

Chronic NLRP3 activation in microglia not only drives IL-1β release but also imprints maladaptive epigenetic programs, including hypomethylation of inflammatory promoters that sustain reactivity. Such “trained” states perpetuate neurotoxic signaling, impair neurogenesis, and foster astrocytic dysfunction [287,288,289]. Inhibitors like MCC950 and next-generation brain-penetrant compounds can disrupt this loop, dampening acute cytokine production while gradually reprogramming microglial memory toward a less inflammatory phenotype [289,290,291]. This mechanistic depth extends their value beyond transient blockade, suggesting that inflammasome inhibition may restore a supportive niche by stabilizing microglial identity and relieving epigenetic brakes on neurogenesis [182,292,293].

While brain-penetrant NLRP3 inhibitors hold strong therapeutic promise, challenges remain in balancing efficacy with safety [294]. Risks include off-target immunosuppression, uncertain timing of intervention, and limited knowledge of long-term effects [294]. Refining specificity through next-generation scaffolds, selective inflammasome modulators, and combinatorial, biomarker-guided strategies could mitigate these concerns [226]. Ultimately, NLRP3 inhibition represents one of the most tangible near-term pharmacological routes to rejuvenating the neurogenic niche, translating mechanistic insights on inflammasome-driven pathology into clinically actionable therapies for aging and neurodegeneration [295].

### 4.5. CRISPR-Based Epigenetic Editing

CRISPR-based epigenetic editing harnesses catalytically inactive Cas9 (dCas9) fused to effector domains that alter chromatin or DNA methylation, enabling locus-specific regulation of gene expression without introducing double-strand breaks [227] (Figure 3E). This distinguishes it from conventional genome editing by allowing reversible, non-mutagenic interventions [227]. For example, dCas9-DNMT3A or DNMT3A/3L fusions can deposit methylation at promoters to silence inflammatory genes, whereas dCas9-TET1 can induce targeted demethylation to reactivate silenced loci such as Oct4 or Fgf21 [228]. Additional configurations, including KRAB- or Ezh2-dCas9 fusions, deposit repressive histone marks, while SunTag-TET systems amplify demethylase recruitment for strong activation [229]. Together, these tools offer unprecedented precision in modulating immune and neurogenic pathways at the epigenetic level [227].

CRISPR-based epigenetic editing directly addresses Gap 2 by enabling the reversal of maladaptive methylation states in neural and immune cells shaped by aging or chronic inflammation [228]. For example, targeting dCas9-DNMT3A to the IL1β promoter in aged microglia could restore silencing through re-methylation, thereby reducing chronic inflammatory drive [230]. Conversely, dCas9-TET1 applied to neurogenic loci such as BDNF or Oct4 can relieve age-induced repression and reactivate transcription, reinstating neurogenic potential in stem cells [228]. Proof-of-concept studies with Yamanaka factors or partial reprogramming confirm that rejuvenating epigenetic marks restores neurogenesis and cognitive capacity in aged niches [266]. Unlike transient cytokine blockade, this strategy reprograms cellular memory itself, offering durable restoration of youthful transcriptional states and opening new avenues for neuroregenerative therapy [231].

Preclinical studies highlight CRISPR-based epigenetic editing as a versatile platform to reshape neuronal and immune gene expression without introducing DNA breaks [232]. In tauopathy models, dCas9-p300 activation of Gad1 restored synaptic inhibition and cognition, while targeted methylation of the APP promoter in Alzheimer’s mice reduced amyloid pathology and memory decline. CRISPRoff approaches have even created heritable transcriptional memory, demonstrating sustained regulation across divisions [232]. In immune cells, epigenetic reprogramming stabilized lineage-specific expression, underscoring durability [296]. Hypothetically, maintaining neurotrophin expression in aged neural stem cells or silencing astrocytic inflammatory mediators could rejuvenate neurogenic niches [297]. By enabling precise, durable, and programmable control of maladaptive states, CRISPR epigenetic editing directly addresses Gap 5, offering a forward-looking strategy to translate mechanistic insight into therapies for neurodegeneration and cognitive decline [232].

Translating CRISPR-based epigenetic editing into the brain faces formidable challenges, with delivery standing as the most immediate hurdle [298]. Viral vectors such as adeno-associated virus (AAV)s provide durable expression but risk insertional mutagenesis and immunogenicity, while nonviral platforms like nanoparticles, nanocapsules, and engineered peptide coatings promise safer, localized delivery yet remain under development [298]. Equally pressing is the need to ensure locus specificity, as off-target chromatin remodeling could introduce unpredictable and durable effects [229,230,232]. Despite these risks, incremental advances in vector design and precision editing suggest that durable, brain-targeted interventions are attainable [298]. In the long term, CRISPR epigenetic editing may become a transformative therapeutic modality, capable of permanently resetting maladaptive cellular states, rejuvenating neural stem cell potential, and sustaining neurogenesis well into aging [299] (Table 3).

## 5. Comparative Perspectives: Human vs. Animal Models

**Figure 4:**
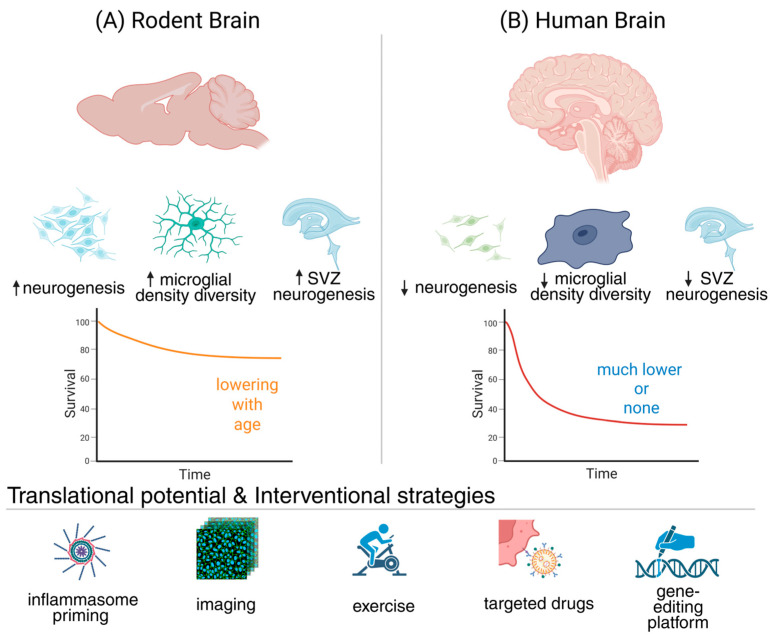
Comparative overview of neurogenic capacity in rodents and humans. Cross-species comparisons are complicated by age-matching and sampling context: many rodent datasets derive from young adult animals, whereas human evidence often relies on post-mortem tissue from older individuals with variable comorbidity and peri-mortem factors. Accordingly, the schematic emphasizes directional trends and methodological uncertainty, not direct quantitative equivalence of neurogenic ‘capacity’ between species. (**A**) Rodent Brain: Rodents maintain relatively high levels of adult neurogenesis across the lifespan, supported by diverse microglial populations and sustained subventricular zone (SVZ) neurogenic activity. This includes robust DG and SVZ neurogenesis in young adulthood and measurable persistence into aging, consistent with preclinical neurogenic literature [1,2,3]. Rodent microglia show increased phenotypic diversity and clear age-associated priming [116,165]. Survival curves illustrate the well-documented gradual decline in neuronal persistence with age, while maintaining observable neurogenic capacity. (**B**) Human Brain: Humans exhibit markedly reduced adult neurogenesis, diminished microglial diversity, and limited or absent SVZ-derived neuronal production. Human studies report much lower baseline neurogenic output, debated persistence in older age groups, and restricted microglial heterogeneity compared with rodents [1,2]. Human microglial transcriptional profiles also show less diverse aging trajectories relative to rodent profiles [304]. The survival curve reflects minimal or near-zero long-term survival of adult-born neurons across adulthood. Translational potential & interventional strategies: The lower panel highlights mechanistic targets and candidate interventions with therapeutic relevance. Inflammasome priming inhibitors: Supported by preclinical NLRP3-blocking agents such as NT-0796, MCC950, BGE-102 [221,222,223,224,226] for longitudinal neuroimmune monitoring: FLT-PET, TSPO-PET, and hybrid PET/MRI [216,217,250]. Exercise-driven neurogenic enhancement: Rodent and translational exercise effects on neurogenesis and inflammatory pathways [301]. Targeted immunopharmacology: Cytokine-level immunomodulation from IL-1β/TNF-driven models [24,29]. Gene-editing platforms: CRISPR-based epigenetic or transcriptional tuning tools [227,229,230,231,232]. SVZ, subventricular zone. Created in BioRender. Tanaka, M. (2026) https://BioRender.com/eqw4kap.

Understanding how rodent and human data align—or diverge—is essential for evaluating the translational relevance of neurogenesis and neuroinflammation research [95]. Animal models provide mechanistic precision, offering evidence for persistent but declining neurogenic activity and for microglial shifts that shape brain plasticity across the lifespan [207]. Human studies, however, reveal greater uncertainty, complicated by methodological variability and ethical constraints [303]. By contrasting these perspectives, we can identify both the strengths and limitations of each approach, setting the stage for a closer examination of adult hippocampal neurogenesis across species [95] (Figure 4).

### 5.1. Adult Neurogenesis: Rodents vs. Humans

Adult rodent studies have firmly established that hippocampal neurogenesis is robust in youth and declines with age, yet it never disappears entirely [82]. Bromodeoxyuridine labeling and lineage tracing demonstrate that new granule cells continue to be generated in the DG, although proliferation rates drop dramatically with aging, from nearly three percent of granule cells in young adults to less than half a percent in old animals [305]. Even under stressors such as ischemia or stroke, aged rodents retain the capacity for injury-induced neurogenesis, with locally activated neural stem and progenitor cells emerging in peri-infarct regions, although their efficiency and neuronal differentiation are reduced [306]. Complementary observations in human stroke and ischemic pathology indicate that neurogenic progenitors and stem-like cells can also be mobilized within or adjacent to injured areas, suggesting that endogenous stem cell pools remain at least partly reprogrammable and capable of contributing to structural repair, even in the aged brain [279,281]. These findings confirm a persistent, though diminished, neurogenic reservoir across the lifespan [82].

In humans, evidence for adult hippocampal neurogenesis remains strikingly divided [307]. Boldrini and colleagues reported thousands of immature neurons persisting even in older adults, whereas Sorrells and collaborators argued that new neurons are virtually absent beyond childhood [308]. Much of this divergence stems from methodological factors: antigen preservation, fixation times, and tissue sampling critically determine whether markers like doublecortin (DCX) or polysialylated neural cell adhesion molecule (PSA-NCAM) are detectable [91]. Reviews emphasize that small differences in processing can yield opposite conclusions, making consensus elusive [94]. The debate continues, with most agreeing that technical rigor, standardized protocols, and multimodal approaches are essential to resolve this controversy [309].

### 5.2. Microglial States Across Species

Rodent studies have provided a detailed atlas of microglial aging, revealing consistent transcriptional and metabolic shifts that define an “inflammaging” signature [310]. Single-cell RNA sequencing across the mouse lifespan uncovers multiple microglial states, with aging marked by heightened chemokine expression and reprogramming of metabolic pathways [165]. A particularly striking feature is the emergence of lipid droplet–accumulating microglia, which display defective phagocytosis, exaggerated cytokine release, and altered lipid metabolism [11]. Proteomic and transcriptomic analyses further demonstrate reduced homeostatic signaling, increased glycolysis, and overlap with disease-associated microglia (DAM) [311]. Collectively, these findings establish aged rodent microglia as pro-inflammatory, metabolically reprogrammed, and primed for maladaptive responses to stress or injury [310].

Human microglia display both striking overlaps with rodents and distinct aging trajectories that underscore species divergence [312]. Transcriptomic studies reveal that while a conserved core program exists, humans show unique regulation of adhesion, cytoskeletal, and complement-related genes, alongside greater transcriptional heterogeneity with age [205]. Unlike rodents, aged human microglia often develop dystrophic morphologies and altered responses to neurodegeneration [101]. Yet shared features emerge: chronic systemic inflammation accelerates microglial aging across species, and both mice and humans exhibit increased T cell infiltration in the SVZ zone, reshaping the neurogenic niche [313]. These parallels and divergences highlight the importance of comparative perspectives for translational relevance (Figure 4A).

### 5.3. Inflammatory Pathways and Neuroimmune Crosstalk

Rodent studies have revealed how inflammasome priming and glial crosstalk shape neurogenic outcomes in aging and disease [314]. Activation of the microglial NLRP3 inflammasome drives the conversion of astrocytes into complement-high reactive programs (often termed “A1-like” in rodent literature, with acknowledged limits to this binary), suppressing neurogenesis and impairing cognition, while genetic deletion of Nlrp3 or treatment with inhibitors such as MCC950 restores function [315]. Similarly, interferon-gamma (IFN-γ)–primed microglia impair neural stem cell proliferation, an effect reversible by janus kinase/signal transducer and activator of transcription 1 (JAK/STAT1) blockade [30]. Tri-culture models confirm that microglia–astrocyte interactions amplify inflammatory cascades, while mitochondrial dysfunction further exaggerates NLRP3 activity [130]. These findings underscore how precisely manipulable rodent systems delineate pathways where inflammation curtails hippocampal neurogenesis.

In humans, evidence for inflammasome activation is largely indirect, derived from postmortem analyses, CSF biomarkers, and emerging imaging studies [316]. Elevated IL-1β, IL-18, and inflammasome proteins such as ASC and caspase-1 have been reported in neurodegenerative disease and TBI, often correlating with severity or outcome [317]. Immunohistochemistry shows co-localization of NLRP3 with glial markers in Alzheimer’s tissue, while iPSC-derived microglia link genetic risk factors to inflammasome priming [318]. Yet interpretation is complicated by timing, chronic disease progression, and comorbidities, making causal inference far less straightforward than in controlled rodent experiments [316] (Figure 4B).

### 5.4. Intervention Efficacy and Translational Readiness

Rodent studies provide strong evidence that lifestyle and experimental interventions can enhance neurogenesis and preserve cognition well into aging and disease [319,320]. Aerobic exercise reduces microglial inflammasome activity through irisin signaling, restoring hippocampal neurogenesis and memory in Parkinson’s models [301]. Environmental enrichment, with or without exercise, consistently improves learning and reduces anxiety-like behaviors, while also limiting aberrant neurogenesis after stroke [321]. Mechanistically, these effects arise from reduced inflammation, epigenetic reprogramming, and enhanced plasticity across hippocampal subregions [322]. Direct manipulations, such as BDNF overexpression or sodium lactate administration, reproduce the benefits of enrichment and exercise, underscoring their causal link to neurogenesis and cognitive resilience [323].

In humans, lifestyle interventions such as exercise, cognitive engagement, and diet consistently improve cognition and brain health, yet their link to neurogenesis remains indirect, inferred from changes in neuroplasticity and neurotrophic signaling rather than direct cellular evidence [324]. Clinical trials of non-steroidal anti-inflammatory drugs (NSAD) in AD have been disappointing, with large-scale meta-analyses showing no meaningful benefit and even highlighting adverse events [325]. This discrepancy with epidemiological associations underscores the complexity of timing and target specificity in human disease [326]. More selective approaches, particularly NLRP3 inflammasome inhibitors, represent an emerging avenue with stronger mechanistic rationale, but translation is still in its infancy, awaiting proof of efficacy and safety in controlled human trials [294].

### 5.5. Bridging the Gap: Models, Ethics, and Future Outlook

Rodent models permit invasive manipulations such as ablation, lineage tracing, and precise genetic editing, approaches that are fundamentally restricted in humans due to ethical and practical barriers [327]. Human studies instead rely on observational designs, neuroimaging, and pharmacological interventions, offering indirect but clinically relevant insights [328]. Post-mortem tissue provides essential molecular detail but also captures late-stage and peri-mortem confounds. Complementing this, living human brain tissue obtained during neurosurgery enables direct assessment of microglial morphology and behavior in viable adult tissue, providing a rare bridge between rodent dynamics and human reality. Incorporating these datasets should sharpen how we interpret ‘dystrophy,’ surveillance, and contact behavior in human microglia [329]. While nonhuman primates and organoid-based chimeras help bridge gaps, their use is also constrained by ethical scrutiny and feasibility [330,331]. Consequently, translational progress depends on integrating mechanistic detail from animal work with non-invasive, ethically sound human research strategies that refine, reduce, and replace invasive experimentation where possible [332].

Future progress will hinge on combining advanced imaging and circulating biomarkers with humanized models that integrate microglia into brain organoids [333]. Such systems allow dynamic visualization of neuron–glia crosstalk, capture human-specific inflammatory signatures, and provide a platform for testing therapeutic strategies [334]. Rodent models remain indispensable for dissecting mechanisms and enabling invasive manipulations that cannot be performed in humans [335]. Yet innovation must increasingly center on organoid-based and biomarker-driven approaches, ensuring translation captures the complexity of human neuroimmunity while retaining the mechanistic clarity offered by animal studies (Figure 4, Table 4).

## 6. Integrating Mechanisms with Therapeutics: Toward Rewiring the Aging Brain

The convergence of mechanistic insight with therapeutic innovation marks a critical frontier in efforts to reshape the course of brain aging [336,337]. Rather than viewing neurogenesis decline and neuroinflammation as inevitable hallmarks of senescence, emerging work reveals them as modifiable processes that can be recalibrated through precise interventions [21]. By aligning detailed knowledge of inflammasome signaling, microglial states, and epigenetic regulation with translational tools such as advanced imaging, targeted drugs, and gene-editing platforms, we begin to chart a roadmap for reprogramming resilience. This section considers how these once-disparate strategies may synergize, offering realistic short-term goals and bold long-term visions for delaying or even reversing cognitive decline [338,339].

### 6.1. Mechanistic Gaps as Opportunities

Gaps in translation should not be seen as obstacles but as navigational markers guiding innovation [340]. Comparative work between animal models and humans consistently reveals mismatches in pathology, timing, and response, yet these very mismatches highlight where new discoveries can be made [341]. Limitations in rodents have prompted the development of humanized organoids, large animal models, and network-based approaches that better capture human complexity [342]. In this way, every identified gap points to a therapeutic or conceptual opportunity, and the strategies that follow can be viewed as direct responses to these translational signposts [343].

Microglial heterogeneity (Gap 1) offers more than complexity; it provides a framework for designing region-specific and state-selective interventions [47]. Rodent studies have mapped diverse transcriptional states across age and pathology, while human transcriptomics confirm that these subtypes exist but follow distinct trajectories, guiding translational priorities [183,344]. Parallel to this, inflammasome-driven epigenetic regulation (Gap 2) emerges as a fertile ground for therapeutic innovation. NLRP3 inhibitors (Strategy 4.4) and CRISPR-based epigenetic editing (Strategy 4.5) exemplify approaches that can reset maladaptive inflammatory programs [294,345]. Thus, what appears as translational limitation is simultaneously the rationale for precision interventions that reprogram cellular states in aging brain niches [278].

Gap 3 highlights systemic influences as a critical axis where rodent and human data converge [346,347]. Parabiosis studies and controlled inflammatory challenges in mice demonstrate the causal power of circulating factors to accelerate or reverse brain aging, while in humans these influences are tracked indirectly through biomarkers, immune profiling, and neuroimaging [348]. Gap 4 reframes modeling constraints as an impetus for innovation, driving the creation of humanized organoids with microglia or vascular integration [349]. Together, these approaches shrink the translational distance, allowing rodent mechanistic insights to be anchored in human relevance and opening new opportunities for therapeutic discovery [350,351].

Gap 5 represents the pivot from mechanism to medicine, where insights from animal models begin to shape clinical opportunity [343,352]. Rodent studies demonstrate that targeting the NLRP3 inflammasome, boosting neurotrophic signaling, or harnessing epigenetic editing can restore neurogenesis and rescue cognition, inspiring translational efforts such as NLRP3 inhibitors now advancing toward human trials [160]. Lifestyle-based interventions likewise show convergent benefits, even if mechanisms differ across species [353]. Comparative perspectives remind us of limits but also highlight optimism: each gap becomes a guidepost, pointing directly to the therapeutic strategies most likely to rejuvenate neurogenesis and combat cognitive decline [354].

### 6.2. Translational Roadmap

Longitudinal imaging stands at the forefront of translational progress, offering a way to monitor neuroinflammation and neurogenesis non-invasively across time [190]. Advances in PET tracers, from second-generation TSPO ligands to newer targets such as COX and P2X7 receptors, promise higher specificity and functional insight [191]. Complementary multiparametric MRI approaches add spatial and physiological context, enabling integration with PET for richer biomarker panels [254]. Yet, robust clinical translation requires rigorous standardization, multicenter reproducibility, and careful correlation of imaging signals with cognitive outcomes [355]. Establishing validated tracers and harmonized protocols will turn imaging into a reliable bridge between mechanistic insight and therapeutic monitoring [356].

Rodent studies have shown that intranasal delivery of EVs, nanoparticles, or viral vectors can modulate microglial activation and promote neuroprotection, and the challenge now lies in scaling these strategies to non-human primates [302]. Advances in engineered AAVs and synthetic promoters already enable selective targeting of glial populations, while refined capsid variants reduce peripheral exposure and off-target effects [357]. Non-viral systems, such as EVs and lipid nanoparticles, add further flexibility and safety [161]. Critical milestones include demonstrating long-term safety, reproducibility, and circuit specificity, ensuring that reprogramming interventions translate effectively into clinically viable therapies [163,358].

NLRP3 inhibitors are moving from preclinical promise to clinical evaluation, with trials in AD now focusing on safety, dose optimization, and early cognitive outcomes as essential milestones [295]. Small molecules such as MCC950, OLT1177, and JC124, alongside emerging biologics, have consistently reduced neuroinflammation and improved cognition in rodent models, and several are progressing into human testing [160,283,359]. Their success will hinge on demonstrating blood–brain barrier penetration, tolerability, and biomarker validation [286,295]. Looking ahead, combinatorial strategies pairing inflammasome inhibition with lifestyle or behavioral interventions may enhance efficacy and broaden therapeutic relevance [286].

CRISPR-based epigenetic editing is advancing rapidly in preclinical research, offering the unprecedented possibility of rewriting maladaptive molecular memory at specific genomic loci [297]. Current efforts center on delivery strategies, with AAV vectors providing strong CNS transduction and nanoparticles emerging as safer, non-viral alternatives [360]. The key challenges are achieving locus specificity, ensuring durability without irreversibility, and minimizing immune or off-target effects [361]. These tools hold unique promise for modulating inflammatory or neurogenic pathways directly at the epigenetic level, yet clinical translation will depend on overcoming safety, delivery, and regulatory hurdles with rigorous precision [297].

The future of translation lies in tailoring interventions to individual neuroinflammatory and epigenetic landscapes, moving beyond one-size-fits-all approaches [362]. Stratifying patients through biomarker and pharmacogenomic profiling could guide the choice of pharmacological, behavioral, or gene-based strategies, while combinatorial therapies may unlock greater synergy than single modalities [363]. Lifestyle programs could complement NLRP3 inhibition or epigenetic editing, and regenerative tools may be personalized for niche restoration [364]. The roadmap remains incremental, yet each milestone brings us closer to clinically rejuvenating neurogenesis and sustaining cognition across aging and disease [203,237].

### 6.3. Ethical and Clinical Considerations

Precision epigenetic editing holds transformative therapeutic potential, yet it brings significant safety challenges that cannot be overlooked [365]. CRISPR-based approaches risk unintended off-target modifications, unpredictable durability of changes, and immune responses triggered by delivery vectors such as AAV or nanoparticles [366]. In the central nervous system, even small errors may have lasting effects, raising concerns about circuit stability and tumorigenesis [367]. Long-term surveillance will be essential to detect delayed consequences of editing [368]. Preclinical pipelines must therefore prioritize reversible, temporally controlled systems and comprehensive off-target profiling before these strategies advance into first-in-human trials [369].

Glial reprogramming represents one of the most exciting frontiers in regenerative neuroscience, yet its clinical translation is shadowed by profound safety concerns [370]. Converting glia into neurons carries risks of aberrant network activity, seizure induction, or tumorigenesis if new cells fail to integrate correctly [269]. Proper synaptic incorporation and maintenance of circuit balance are therefore paramount, as incomplete or uncontrolled conversion could destabilize neural networks [371]. The promise of replacing lost neurons must be weighed against an ethical duty to protect vulnerable patients, ensuring that regenerative enthusiasm does not outpace rigorous safety and ethical oversight [278].

Immune modulation in the aging brain presents a profound double-edged challenge. While suppressing microglial overactivation or dampening inflammasome signaling may restore plasticity and cognitive resilience, excessive suppression risks compromising pathogen defense, elevating vulnerability to infection or even cancer [372]. Calibrating these interventions in elderly patients therefore requires a fine balance between rejuvenation and safety [372]. Stratification by immune competence, genetic background, and comorbidities will be critical for minimizing harm [373]. Ultimately, personalized immune interventions must progress cautiously, ensuring that therapeutic innovation aligns with the biological realities of aging immunity.

Preventive interventions in normal aging raise complex ethical dilemmas, particularly when the therapies under consideration carry high risks [374]. At what threshold does delaying cognitive decline justify invasive gene editing or immune modulation in individuals without disease? Autonomy and informed consent must remain central, yet both are challenged by uncertainty in predicting benefit [374]. Societal concerns also arise, from medicalizing normal aging to reinforcing inequities in access [374]. Defining clear thresholds of clinical risk versus potential gain is therefore essential before preventive neuroenhancement can be ethically endorsed.

Regulatory pathways in neurodegenerative interventions differ markedly, with small molecules such as NLRP3 inhibitors often advancing more rapidly than gene or cell-based therapies, which require complex oversight [294]. Designing trials that capture both safety and meaningful outcomes remains essential [375]. Beyond adverse events, endpoints must include cognition, neurogenesis biomarkers, and quality of life to demonstrate true clinical relevance [375]. Harmonized protocols, long-term monitoring, and adaptive trial designs will be critical [376]. Ultimately, ethical rigor and regulatory foresight provide the scaffolding for translating scientific breakthroughs into safe, responsible therapies [377].

## 7. Conclusions

Neurogenesis and neuroinflammation exist in continuous dialogue, shaping how the brain ages and responds to stress [12]. This review has highlighted that disrupting this dialogue accelerates decline, while recalibrating it can preserve or even restore cognitive resilience. Mapping key gaps and aligning them with targeted strategies provides a roadmap for intervention [2]. The central message is clear: the neurogenic potential of the aging brain is not lost but remains accessible if the immune environment is carefully tuned. Even in late life, glia and neural stem cells retain remarkable plasticity. Harnessing this latent capacity requires converging approaches, from modulating microglial states and inflammasome signaling to applying epigenetic editing, gene therapy, and novel imaging biomarkers [378,379]. These strategies are more than incremental advances; they represent a shift in how we conceptualize and attempt to reshape the brain’s fate during aging. The novelty and significance of these approaches lie in their integrative scope. By combining mechanistic insights with innovative technologies, we are beginning to see the contours of interventions that could delay neurodegeneration and protect cognition. The challenge now is translation: embedding safety, ethics, and rigorous trial design into every step. With continued interdisciplinary collaboration, the prospect of actively guiding the neuroimmune dialogue toward healthier brain aging is within reach [2,222].

## Figures and Tables

**Table 1:** Molecular pathways linking inflammation and neurogenesis. Molecular pathways connecting inflammation and adult neurogenesis, summarizing key cytokines, chemokines, neurotrophic factors, and intracellular signaling hubs relevant to stem cell regulation in the adult brain. Each entry outlines the primary cellular source, directional impact on neural stem cell proliferation, differentiation, or survival, age-associated expression shifts, and potential therapeutic interventions or pharmacological modulators targeting the listed molecule or pathway [159]. References include both primary experimental studies and integrative reviews supporting each pathway (e.g., [146,147,148,149,150]). Examples include IL-1β (microglial-derived negative regulator), TNFα (context-dependent neurogenic suppressor), CX3CL1/CX3CR1 (microglia–neuron communication axis), IGF-1 and BDNF (trophic pro-neurogenic drivers), as well as inflammasome nodes such as NLRP3. This reference table aims to support target discovery, translational prioritization, and mechanistic modeling across neuroinflammation-neuroplasticity research.

Molecule/Pathway	Source/Cell Type	Effect on Neurogenesis	Relevance in Aging	Targeted by	References
IL-1β	Activated microglia	Inhibits NSC proliferation and newborn neuron survival; blocks maturation	Chronically elevated with NF-κB/NLRP3 activation; contributes to hostile niche	NLRP3 inhibitors (MCC950, NT-0796), anti-IL-1 drugs	[24,29,30]
TNF-α	Activated microglia	Suppresses progenitor proliferation and neuronal differentiation	Increased in microglial ‘primed’ states during inflammaging	TNF pathway blockers	[24,29]
IL-6	Activated microglia/astrocytes	Reduces NSC proliferation; impairs plasticity	Elevated with chronic NF-κB/NLRP3 signaling	Anti-IL-6 agents (exploratory)	[24,29]
IFN-γ	Infiltrating CD8^+^ T cells; activated microglia	Suppresses NSC proliferation; antineurogenic bias	T-cell accumulation in aged niches; drives microglial priming	JAK/STAT inhibitors	[30]
NLRP3 inflammasome	Microglia	Sustains IL-1β/IL-18; locks antineurogenic programs	Persistently activated in aging; imprints epigenetic ‘scars’	Brain-penetrant NLRP3 inhibitors	[29,151]
NF-κB	Microglia/astrocytes	Pro-inflammatory transcription; suppresses neurogenesis	Chronically active with oxidative stress; feeds cytokine loop	Pathway modulators (research)	[29,151]
Complement (C1q/C3)	Microglia/astrocytes	Accelerated pruning; survival loss of newborns	Heightened with chronic inflammatory tone	Complement inhibitors	[31,153]
CX3CL1–CX3CR1	Neurons → microglia	Maintains microglial quiescence; supports maturation/integration	Protective tone wanes with age; disruption impairs neurogenesis	CX3CR1/CX3CL1 agonists	[28,152,154,158]
IGF-1	Microglia, niche cells	Promotes NSC proliferation and survival	Declines with aging; part of youthful pro-neurogenic secretome	IGF-1 delivery/mimetics	[26,27]
BDNF/TrkB	Microglia, neurons	Enhances proliferation, maturation, survival; plasticity	Reduced availability under chronic inflammation	TrkB agonists; BDNF delivery	[26,27]
TGF-β	Microglia/astrocytes, niche	Context-dependent; supports homeostasis in youth	Elevated tonic signaling with age constrains neurogenesis	TGF-β tuning (local)	[26,27]
IL-10	Microglia/astrocytes	Pro-neurogenic, supports integration	Protective signals decline with age	Cytokine augmentation	[35]
PI3K–Akt/ERK/Wnt–β-catenin	NSCs; microglia-modulated	Downstream pro-neurogenic cascades	Suppressed under inflammatory milieu	Small-molecule activators	[26,149]
CD8^+^ T-cell entry	Peripheral T cells	IFN-γ-mediated suppression of NSCs	Accumulate in aged SGZ/SVZ; feed-forward loop	Blockade of entry/adhesion	[30]

Akt, Protein Kinase B (PKB); BDNF, Brain-Derived Neurotrophic Factor; C1q, Complement Component 1q; C3, Complement Component 3; CD8^+^, Cluster of Differentiation 8-Positive T Cell; CX3CL1, Chemokine (C-X3-C Motif) Ligand 1; CX3CR1, Chemokine (C-X3-C Motif) Receptor 1; ERK, Extracellular Signal-Regulated Kinase; IFN-γ, Interferon-Gamma; IGF-1, Insulin-Like Growth Factor-1; IL-1β, Interleukin-1 Beta; IL-6, Interleukin-6; IL-10, Interleukin-10; JAK/STAT, Janus Kinase/Signal Transducer and Activator of Transcription; MCC950, NLRP3 Inflammasome Inhibitor (Small Molecule); NF-κB, Nuclear Factor Kappa-Light-Chain-Enhancer of Activated B Cells; NLRP3, NOD-Like Receptor Pyrin Domain-Containing Protein 3; NSC, Neural Stem Cell; NT-0796, Brain-Penetrant NLRP3 Inhibitor; PI3K, Phosphoinositide 3-Kinase; SGZ, Subgranular Zone; SVZ, Subventricular Zone; TGF-β, Transforming Growth Factor-Beta; TNF-α, Tumor Necrosis Factor-Alpha; TrkB, Tropomyosin Receptor Kinase B; Wnt, Wingless-Related Integration Site.

**Table 2:** Five key knowledge gaps in neurogenesis–neuroinflammation. Five key knowledge gaps linking neurogenesis and neuroinflammation, summarizing unresolved questions, current unknowns, biological and clinical relevance, and candidate methodological strategies. Columns include: Gap, Description of Unknown, Why it Matters/Potential Consequences, and Suggested Approaches. Representative entries may include regional microglial specialization, age-dependent inflammatory plasticity, inflammasome–neurogenesis coupling, long-term effects of transient immune activation, and sex-specific neuroimmune interactions. This table provides a rapid reference to complement Section 3 and guide hypothesis formulation, experimental design, and translational priority setting.

Gap	Description of Unknown	Why It Matters/Consequences	Suggested Approaches	References
1. Regional Microglial Diversity	Limited understanding of how microglial phenotypes differ across brain regions and influence neurogenesis	Regional vulnerabilities exist (hippocampus vs. olfactory bulb); lack of clarity hampers targeted interventions	Single-cell RNA-seq, region-specific lineage tracing, conditional microglial manipulation	[132,133,134]
2. Inflammasome Dynamics in Aging	Unresolved timeline of NLRP3/other inflammasome activation in aged niches	Unclear when inflammasome priming becomes irreversible; timing critical for therapeutic window	Longitudinal transcriptomics, in vivo biosensors, inducible knockout models	[130,131,160]
3. Crosstalk Between Peripheral and CNS Immunity	Mechanisms of how peripheral T cells and cytokines reshape neurogenic niches remain obscure	Infiltrating T cells alter NSC fate; missing mechanistic detail limits translation to systemic therapies	Fate-mapping of immune infiltration, parabiosis, targeted blockade of adhesion molecules	[132,133,134]
4. Beneficial vs. Detrimental Microglial States	Poorly defined markers distinguishing pro-neurogenic vs. antineurogenic microglial states	Current therapies risk indiscriminate immunosuppression; need precision immunomodulation	Multi-omics integration (proteome, epigenome), machine-learning-based state classification, microglia-specific drug screens	[5,6,7]
5. Non-coding RNA & Extracellular Vesicle Signaling	Roles of EV cargo (miRNAs, lncRNAs) in regulating neurogenesis under inflammation are underexplored	Missed therapeutic opportunities; EVs may carry both detrimental and reparative signals	High-resolution EV profiling, CRISPR-based RNA manipulation, engineered EV delivery systems	[161,162,163]

CNS, Central Nervous System; EV, Extracellular Vesicle; lncRNA, Long Non-Coding RNA; miRNA, MicroRNA; NLRP3, NOD-Like Receptor Pyrin Domain-Containing Protein 3; NSC, Neural Stem Cell; RNA-seq, RNA Sequencing.

**Table 3:** Emerging therapeutic strategies targeting the neuroimmune axis. Overview of emerging therapeutic strategies aimed at modulating the neuroimmune axis to protect or restore adult neurogenesis, outlining representative tools, intended biological effects, and current translational maturity. Columns include: Strategy, Examples/Tools, Goal/Effect, and Stage of Development. Representative entries may feature longitudinal neuroimmune imaging modalities (e.g., [^18F]FLT PET, TSPO-PET), brain-penetrant NLRP3 inflammasome inhibitors (e.g., MCC950, NT-0796) [300], in vivo glial reprogramming vectors (e.g., AAV-NeuroD1), precision epigenetic gene editing platforms (e.g., CRISPR-dCas9), and niche-targeted immunomodulatory therapeutics. This table offers a rapid translational snapshot for investigators evaluating feasibility, clinical readiness, and mechanistic alignment across intervention classes.

Strategy	Examples/Tools	Goal/Effect	Stage of Development	References
Longitudinal Imaging	[^18F]FLT-PET for neurogenesis, TSPO-PET for microglial activation	Enables in vivo monitoring of neurogenesis and neuroinflammation across lifespan	Preclinical for neurogenesis tracers; TSPO-PET in human use	[216,217,218,245,246]
Brain-Penetrant NLRP3 Inhibitors	MCC950, NT-0796, BGE-102	Reduce chronic IL-1β release, restore neurogenic potential	Preclinical to Phase 1 clinical trials	[221,222,223,224,225,226]
Glial Reprogramming	AAV-NeuroD1, SOX2-based astrocyte-to-neuron conversion	Replace lost neurons; rejuvenate circuits	Proof-of-concept in rodents	[192,219,273]
CRISPR Epigenetic Editing	CRISPR-dCas9 targeting IL-1β/NLRP3 loci; enhancer repression	Long-term silencing of pro-inflammatory genes without DNA cleavage	Lab-stage; in vitro and early in vivo	[227,228,229,230,231,232]
Niche Immunomodulation	Anti-IL-1β, anti-TNF, IL-6R antibodies; microglia-specific modulators	Dampens chronic inflammation in neurogenic niches	Several agents in AD, MCI, depression trials	[294,295,301]
Extracellular Vesicle (EV) Therapeutics	Engineered EVs carrying miRNAs, BDNF, or IGF-1 cargo	Deliver pro-neurogenic and anti-inflammatory signals	Preclinical; first-in-human safety studies emerging	[161,163,302]
Lifestyle & Activity-Based Interventions	Exercise, enriched environment, caloric modulation	Boost endogenous IGF-1/BDNF, reduce inflammatory priming	Multiple human cohort studies and ongoing clinical trials	[249,301]
Small-Molecule Neurotrophic Enhancers	TrkB agonists, phosphodiesterase inhibitors	Enhance BDNF signaling, promote synaptic/neurogenic resilience	Early-stage clinical testing, mixed outcomes	[216,248]
Microglial State Modulation	CSF1R inhibitors, TREM2 agonists	Shift microglia from pro-inflammatory to reparative states	Preclinical; TREM2 antibodies in Phase 2 AD trials	[217,250]
Combinatorial Approaches	NLRP3 inhibitor + exercise; anti-TNF + BDNF mimetics	Target multiple axes (inflammatory and trophic) simultaneously	Conceptual and early preclinical testing	[221,301]

AAV, Adeno-Associated Virus; AD, Alzheimer’s Disease; BDNF, Brain-Derived Neurotrophic Factor; CSF1R, Colony-Stimulating Factor 1 Receptor; CRISPR, Clustered Regularly Interspaced Short Palindromic Repeats; dCas9, Deactivated CRISPR-Associated Protein 9; EV, Extracellular Vesicle; [^18F]FLT, Fluorothymidine Labeled With Fluorine-18; IGF-1, Insulin-Like Growth Factor-1; IL-1β, Interleukin-1 Beta; IL-6R, Interleukin-6 Receptor; MCI, Mild Cognitive Impairment; miRNA, MicroRNA; NLRP3, NOD-Like Receptor Pyrin Domain-Containing Protein 3; PET, Positron Emission Tomography; SOX2, SRY-Box Transcription Factor 2; TNF, Tumor Necrosis Factor; TrkB, Tropomyosin Receptor Kinase B; TSPO, Translocator Protein 18 kDa.

**Table 4:** Comparative characteristics—neurogenesis and neuroinflammation in mice vs. humans. Side-by-side comparison of core biological, cellular, and aging-related features of adult hippocampal neurogenesis and neuroinflammatory remodeling in rodents versus humans. Columns include: Aspect, Rodents (Murine), and Humans. Representative comparison points may include baseline levels of adult neurogenesis, age-related rates of decline, microglial density and activation phenotypes across lifespan, peripheral immune cell infiltration into the central nervous system, and responsiveness to lifestyle-based interventions such as exercise or environmental enrichment. This table serves as a rapid translational reference, highlighting biological similarities, species-specific divergence, and areas where human data remain limited or method-dependent.

Aspect	Rodents (Murine)	Humans	References
Adult hippocampal neurogenesis (baseline)	Thousands of new neurons per day in young adult hippocampus; robust measurable pools	Far fewer (hundreds/day in young adults by some estimates); highly variable depending on methodology	[1,2,3]
Age of significant decline in neurogenesis	Detectable decline starting mid-life (12–18 months); still measurable in aged animals	Steep decline reported from middle age; ongoing debate whether residual neurogenesis persists in elderly	[1,2,3]
Microglial density and activation state in aging	Well-characterized shift to ‘primed’ phenotype with pro-inflammatory gene expression and reduced phagocytic resolution	Less comprehensive; aged human microglia show pro-inflammatory signatures, distinct subsets identified via single-cell transcriptomics	[116,165]
Peripheral immune cell involvement in CNS with age	Increased infiltration of T cells (especially CD8^+^) into hippocampus and SVZ with aging; enhances IFN-γ tone	Limited but growing evidence; T-cell presence in human hippocampus in aging and neurodegeneration; mechanisms less defined	[132,133]
Evidence for exercise or enrichment effects	Exercise and enriched environments robustly increase neurogenesis and improve cognition in mice	Human studies show hippocampal volume increases and cognitive benefits; direct evidence for neurogenesis boost is indirect (MRI, blood biomarkers)	[216,248,301]
Inflammasome/NLRP3 activation with age	Strong evidence for NLRP3-driven IL-1β increase in aged rodent hippocampus, reducing neurogenesis	Human post-mortem and transcriptomic studies support NLRP3 upregulation in aging brain; functional causality harder to confirm	[221,222,301]
Translational caveats	High plasticity, short lifespan, and controlled environments amplify experimental effects	Human variability, long lifespan, and heterogeneous exposures complicate translation; methodological debates on detecting neurogenesis	[1,2,3]

CD8^+^, Cluster of Differentiation 8-Positive T Cell; CNS, Central Nervous System; IFN-γ, Interferon-Gamma; IL-1β, Interleukin-1 Beta; MRI, Magnetic Resonance Imaging; NLRP3, NOD-Like Receptor Pyrin Domain-Containing Protein 3; SVZ, Subventricular Zone.

## Data Availability

No new data were created or analyzed in this study.

## Abbreviations

The following abbreviations are used in this manuscript:

AD	Alzheimer’s disease
AAV	adeno-associated virus
BBB	blood–brain barrier
BDNF	brain-derived neurotrophic factor
CNS	central nervous system
CRISPR	clustered regularly interspaced short palindromic repeats
CSF	cerebrospinal fluid
CX3CL1	C-X3-C motif chemokine ligand 1
CX3CR1	C-X3-C motif chemokine receptor 1
DAM	disease-associated microglia
DCX	doublecortin
DG	dentate gyrus
EVs	extracellular vesicles
IFN-γ	interferon-gamma
IL-1β	interleukin-1 beta
IL-18	interleukin-18
iPSC	induced pluripotent stem cell
JAK/STAT1	janus kinase/signal transducer and activator of transcription 1
MCC950	nlrp3 inflammasome inhibitor mcc950
MRI	magnetic resonance imaging
NLRP3	nod-like receptor protein 3
NSAID	non-steroidal anti-inflammatory drug
NSPCs	neural stem and progenitor cells
PET	positron emission tomography
PSA-NCAM	polysialylated neural cell adhesion molecule
RNA-seq	RNA sequencing
SVZ	subventricular zone
TBI	traumatic brain injury
TNF-α	tumor necrosis factor-alpha
TSPO	translocator protein 18 kDa
Wnt	wingless-related integration site signaling pathway
